# Stability of Crystal Nuclei of Poly (butylene isophthalate) Formed Near the Glass Transition Temperature

**DOI:** 10.3390/polym12051099

**Published:** 2020-05-11

**Authors:** Silvia Quattrosoldi, Nadia Lotti, Michelina Soccio, Christoph Schick, René Androsch

**Affiliations:** 1 Department of Civil, Chemical, Environmental and Materials Engineering, University of Bologna, Via Terracini 28, 40131 Bologna, Italy; silvia.quattrosoldi2@unibo.it (S.Q.); nadia.lotti@unibo.it (N.L.); m.soccio@unibo.it (M.S.); 2 Interdisciplinary Center for Transfer-oriented Research in Natural Sciences (IWE TFN), Martin Luther University Halle-Wittenberg, 06099 Halle/Saale, Germany; 3 Butlerov Institute of Chemistry, Kazan Federal University, 18 Kremlyovskaya Street, 420008 Kazan, Russia; christoph.schick@uni-rostock.de

**Keywords:** poly(butylene isophthalate), crystallization, nucleation, nuclei stability, Tammann’s nuclei development method, transfer heating rate

## Abstract

Tammann’s two-stage crystal-nuclei-development method is applied for analysis of the thermal stability of homogenously formed crystal nuclei of poly(butylene isophthalate) (PBI) as well as their possible reorganization on transferring them to the growth temperature, using fast scanning chip calorimetry. Crystal nuclei were formed at 50 °C, that is, at a temperature only slightly higher than the glass transition temperature, and developed to crystals within a pre-defined time at the growth temperature of 85 °C. The number of nuclei, overcritical at the growth temperature, was detected as a function of the transfer-conditions (maximum temperature, heating rate) by evaluation of the developed crystal fraction. For different size-distributions of crystal nuclei, as controlled by the nucleation time, there is detected distinct reduction of the nuclei number on heating to maximum temperatures higher than about 90 to 110 °C, with the latter value holding for longer nucleation time. Longer nucleation allows for both increasing the absolute nuclei number and generation of an increased fraction of larger nuclei. Heating at 1000 K/s to 140–150 °C causes “melting” of even the most stable nuclei. While direct transfer of crystal nuclei from the nucleation temperature (50 °C) to the growth temperature (85 °C) reveals negligible effect of the transfer-heating rate, in-between heating to higher temperatures is connected with distinct nuclei-reorganization above 85 °C on heating slower than 1000–10.000 K/s. The performed study not only provides specific valuable information about the thermal characteristics of crystal nuclei of PBI but also highlights the importance of proper design of Tammann’s nuclei development experiment for analysis of nuclei numbers. With the evaluation of critical rates of temperature-change for suppression of non-isothermal formation of both nuclei and crystals, the kinetics of crystallization of the slow crystallizing PBI is further quantified.

## 1. Introduction

Poly(butylene isophthalate) (PBI) is a linear aromatic polyester with a chemical structure similar to the well-known isomer poly(butylene terephthalate) (PBT) [1]. The only difference is the linkage of the phenylene ring with the neighboring ester groups. In case of PBT, the ester groups are in para-position, while in PBI they are in meta-position to each other. This small difference in the chemical structure causes largely different crystallization behavior and affects potential applications. PBT is a rather fast crystallizing polymer, even allowing the generation of semicrystalline products in typical polymer processing routes like injection molding [2,3,4]. The glass transition and equilibrium melting temperatures of PBT are slightly above room temperature [5] and between about 230 and 250 °C [6,7,8], respectively; the temperature dependence of the rate of melt-crystallization shows maxima at around 70 and 145 °C [9,10,11]. These maxima correspond to halftimes of crystallization of the order of magnitude of 0.1 and 1 s, respectively, and are associated with crystallization via homogenous and heterogeneous crystal nucleation, respectively [11,12,13]. PBI exhibits a similar glass transition temperature as PBT [14,15], however the crystals are less stable and exhibit a distinctly lower equilibrium melting temperature, with values reported being between 146 [15] and 165 °C [16]. Melt-crystallization of PBI is fastest between around 80 and 100 °C, with the minimum crystallization halftime being of the order of magnitude of several minutes [17,18]. Such a slow crystallization is a major drawback when attempting to obtain a semicrystalline structure for specific engineering uses through industrial processing routes, and it seemingly now limits its commercial application. This notwithstanding, excellent mechanical behavior, good barrier properties, and easy melt-processability are reported for PBI [19], with ongoing research also including its derivatives, such as end-capped materials, block copolymers, and random copolymers [19,20,21,22,23,24,25,26], for possible application, e.g., as hot-melt adhesive or coating.

Recently, an attempt to modify the crystallization rate and semicrystalline morphology of PBI via controlling the crystal-nucleation pathway was reported [27]. It was found that low-temperature annealing close to the glass transition temperature leads to relatively fast formation of homogenous crystal nuclei, which grow to crystals at elevated temperatures and cause a much finer spherulitic superstructure than is obtained after direct melt-crystallization, with anticipated implications, e.g., on the mechanical or optical properties [28,29,30,31]. Detailed information about the temperature dependence of the nucleation kinetics were provided based on the application of Tammann’s two-stage crystal nuclei development method [27] in order to overcome the problem of the impossible direct detection of homogenous nuclei by imaging or calorimetric methods. Tammann’s method is based on the observation that the temperatures of the maximum nucleation rate and maximum crystal growth rate often are largely different [32,33,34]. It includes in a first step formation of nuclei at a rather low temperature where the crystal growth rate is negligible, that is, at a high supercooling of the melt or even in the glassy state. Subsequently, the nuclei, which form as a function of time at low temperatures, are transferred to a higher temperature to allow their development/growth to detectable sizes. Tammann’s protocol was successfully applied for the analysis of the homogenous nucleation of glycerol [32,33], organic liquids [35], and silicate glasses [36,37], and more recent research proved that it can also be used for polymers. Regarding the latter, quantitative information about the nucleation kinetics was provided for poly (ε-caprolactone) (PCL) [38,39], poly(l-lactic acid) (PLLA) [40,41,42,43], isotactic poly(butene-1) (iPB-1) [44], polyamide 6 (PA 6) [45], and poly(ethylene terephthalate) (PET) [46]. It is worth noting that the technology for producing glass ceramics is based on Tammann’s nuclei-development scheme [47].

Additionally, for PBI, first data about the nucleation behavior were gained using Tammann’s experiment [27], revealing that nucleation is fastest at around 50 °C, with nuclei beginning to form after about 1 s at this temperature. The temperature of fastest nucleation is distinctly lower than the temperature of highest crystal growth rate, being slightly higher than 90 °C, confirming the early observation of Tammann that the temperature dependencies of nucleation and growth of crystals are often largely different. This notwithstanding, the design of the initial nucleation experiments on PBI was rather arbitrary, as the impact of important test parameters was not completely investigated. This holds for the so-called nuclei-transfer heating rate and the definition of the growth-stage temperature of Tammann’s protocol. In the nucleation step, at a pre-defined temperature, nuclei with a specific size-distribution form as a function of the nucleation time. In order to quantify their number, these nuclei need to grow to detectable size at the growth stage. However, at the growth stage, only nuclei of supercritical size at the growth temperature will develop to crystals and smaller nuclei will “melt” on their transfer from the nucleation- to the growth temperature. As such, the growth-stage temperature controls which nuclei of the initial size distribution are detected. The higher the growth temperature is, the smaller the nuclei size fraction is at its large size end, which is probed. It is obvious that variation in the growth-stage temperature allows assessing of the nucleus size distribution. The precondition for the analysis of the above-described link between the nucleus size and the growth-stage temperature is absence of growth of nuclei during the transfer from the nucleation to the growth stage. Similarly to crystal reorganization [48,49,50], heating slower than a critical value allows nuclei growth/stabilization and even nuclei formation, distorting conclusions about the nucleation kinetics at the nucleation stage. Earlier studies about the effect of the growth conditions in Tammann’s experiments focused on lithium disilicate systems [51,52], however first results for polymers also appeared recently [43,53].

In order to accommodate the rather novel information about important effects of the transfer heating rate and growth temperature in Tammann’s experiment on characterizing the nucleation characteristics, the initial crystallization study of PBI [27] is expanded with this work in order to further quantify its nucleation behavior. In the first part, this paper provides information about the critical rates of temperature change, above which the non-isothermal formation of nuclei and crystals is suppressed. This knowledge is required for assuring strict isothermal nuclei formation at the selected pre-defined nucleation temperature of 50 °C in Tammann’s experiment, including avoiding nuclei formation on heating when the nucleation at 50 °C was incomplete. The second part of the study provides data about the thermal stability of the nuclei. Different size-distributions of nuclei, generated at 50 °C by variation of the nucleation time, are heated to different maximum temperatures up to 150 °C, only allowing the survival of nuclei with a size larger than the critical size at the respective maximum temperature. Their number equivalent, that is, the crystal fraction forming within a predefined time, is then detected using a growth temperature of 85 °C. In the third and final part of the study, the effect of the heating rate on the transfer of nuclei from the nucleation temperature of 50 °C to the various maximum temperatures is explored, with low and high heating rate limits defined by the critical rate of temperature change needed to avoid non-isothermal crystallization and instrument characteristics, respectively.

## 2. Materials and Methods

The PBI was synthesized as described in detail elsewhere [27]. The number average molar mass and polydispersity of the polymer were 33.150 g/mol and 2.0, respectively. The as-synthesized material was compression-molded to a film of 100 µm thickness, employing a Carver laboratory press. The polymer was sandwiched between two Teflon™ sheets and then heated to 200 °C. After annealing for 2 min, the sandwich was subjected to a pressure of 5 tons/m^2^ for a further 2 min, removed from the press, and ballistically cooled to room temperature.

Fast scanning chip calorimetry (FSC) was used for the analysis of the kinetics of non-isothermal crystal nucleation, the thermal stability of isothermally formed nuclei, as well as the effect of the transfer heating rate on nuclei reorganization in Tammann’s nuclei development experiment. We used a Flash DSC 1 (Mettler-Toledo, Greifensee, Switzerland) connected to an intracooler TC100 (Huber, Offenburg, Germany). The specimen preparation included cutting of thin sections with a thickness of 10 µm using a rotary microtome (Slee medical GmbH, Mainz, Germany) and reducing the lateral size of the sections to about 50 to 100 µm employing a stereomicroscope. Before loading the sample onto the membrane of the UFS 1 sensor, the latter was conditioned and temperature-corrected using predefined routines in the instrument software. The sample environment was purged with nitrogen gas at a flow rate of around 40 mL/min, and the sensor support temperature was set to −90 °C. Detailed information about temperature–time profiles is provided below.

## 3. Results and Discussion

### 3.1. Critical Rates of Temperature-Change for Prevention of Non-Isothermal Crystal Nucleation and Crystal Growth

**Figure 1:**
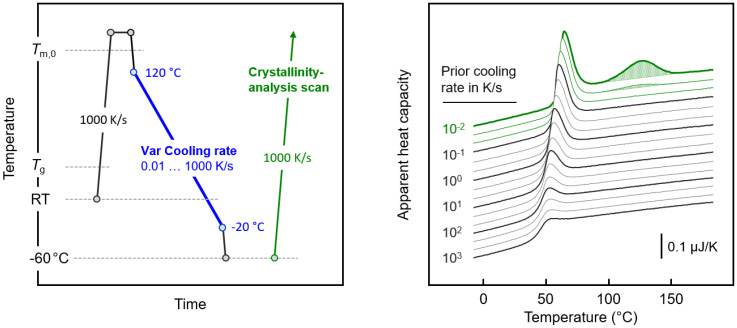
Temperature–time profile for the analysis of the cooling rate dependence of the crystallization of poly(butylene isophthalate) PBI (**left**), and fast scanning chip calorimetry (FSC) heating scans for the analysis of the formation of crystals during prior cooling at rates of 1000, 500, 200, 100, 50, 20, 10, 5, 2, 1, 0.5, 0.2, 0.1, 0.05, 0.02, and 0.01 K/s (from bottom to top) (**right**).

Pre-requisite for the analysis of the stability of crystal nuclei is the knowledge of the condition for their controlled isothermal formation. It requires cooling the melt at a rate that will not allow nuclei formation nor crystallization before reaching the nucleation temperature. In order to obtain the critical cooling rate below which crystallization occurs, the sample was subjected to the temperature–time profile shown in the left plot of Figure 1. As such, PBI was cooled at rates between 0.01 and 1000 K/s (blue segment), and then the crystal fraction formed during cooling was analyzed on subsequent heating via the enthalpy of melting (green segment). Note that the variation of the cooling rate was limited to the temperature range below 120 °C, for the sake of reducing the time interval of keeping the sample in a molten state and avoiding risking polymer degradation. Below −20 °C, the cooling rate variation was assumed not to affect the structure formation, as at temperatures lower than 50 K below *T*_g_ cooperative segmental mobility is absent. The right plot of Figure 1 shows analysis heating scans recorded after subjecting the sample to cooling at different rates, as indicated at the left-hand side of the various curves. Accordingly, cooling at rates higher than 0.02 K/s does not allow crystallization, as only the glass transition, superimposed by an enthalpy recovery peak, is detected at around 50 °C. The first trace of a melting peak is seen for the sample cooled at 0.02 K/s, with the peak emphasized by shading. Cooling at a rate lower than 0.01 K/s further increases the crystallinity, as concluded from the larger melting peak recorded in the heating scan. Though much lower cooling rates are needed to adjust the maximum crystallinity, the data of Figure 1 revealed a rather low critical cooling rate of 0.05 K/s (0.3 K/min) to completely suppress crystallization.

**Figure 2:**
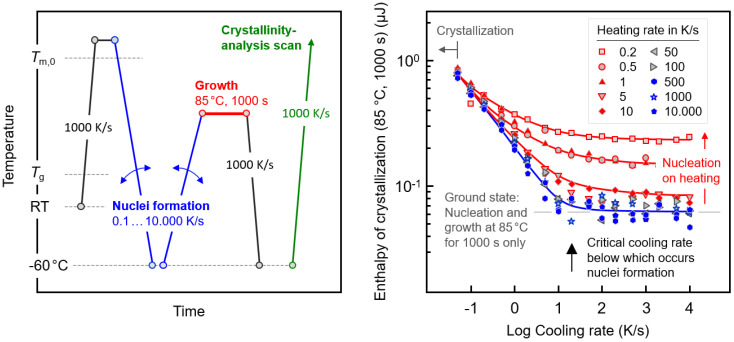
Temperature–time profile for the analysis of the cooling and heating rate dependencies of crystal nucleation of PBI (**left**); enthalpies of crystallization at 85 °C for 1000 s after subjecting the polymer to different cooling and heating histories (**right**). The lines serve to guide the eye only.

Figure 2 introduces an experiment for obtaining the critical rates of cooling and heating to suppress not only crystallization but also nucleation. The left plot shows the temperature–time profile imposed on the sample. The equilibrium melt is cooled through the crystallization-relevant temperature range and then re-heated to 85 °C, both at systematically changed rates between 0.01 and 10,000 /Ks (blue segments). At 85 °C, the sample subjected to a specific cooling and reheating history is isothermally annealed with the purpose of growing the eventually formed nuclei into crystals within 1000 s (red segment). The amount of crystals formed at these fixed-growth conditions is then analyzed via the enthalpy of their melting in the final crystallinity analysis heating scan (green segment), shown as a function of the cooling rate (abscissa) and heating rate (see legend) in the right plot of Figure 2. The experiment of Figure 2 resembles Tammann’s two-stage nuclei development experiment, allowing for separate nucleation and growth steps. It is anticipated that cooling and re-heating allows for the formation of increasing numbers of nuclei when reducing the rates of cooling or heating; however, it assures the absence of crystal growth as long as the minimum rate of temperature change is higher than 0.1 K/s (see also Figure 1). The number of nuclei formed during cooling or heating then controls the crystal fraction at the pre-defined growth condition.

The data of the right plot of Figure 2 reveal that on very fast heating of the system, as represented/approximated by the blue-colored pentagons obtained using a heating rate of 10,000 K/s, crystal nuclei form in the prior cooling segment if the cooling rate is lower than about 20 K/s (see the black arrow at the cooling rate axis). On cooling at rates lower than 20 K/s, the enthalpy of crystallization increases to above the ground level, indicated with the gray horizontal line. The ground state represents the enthalpy of crystallization at 85 °C for 1000 s after rapid cooling the equilibrium melt to the growth stage temperature, that is, without in-between nuclei formation on cooling to a lower temperature and heating. In other words, if the melt is cooled at 20 K/s or lower rates, then nuclei form on cooling and accelerate the crystallization at 85 °C. Similarly, on very fast cooling, e.g., 10,000 K/s, not allowing nuclei formation, an increase in the crystallinity is detected if the heating rate is lower than 50 K/s (see red-colored data sets and red vertical arrow to the right of the curves). As such, the main result of the experiment in Figure 2 is the quantification of the critical rates of cooling and heating of around 20 K/s, above which there is no crystal nuclei formation.

### 3.2. Thermal Stability of Crystal Nuclei Formed Near the Glass Transition Temperature

**Figure 3:**
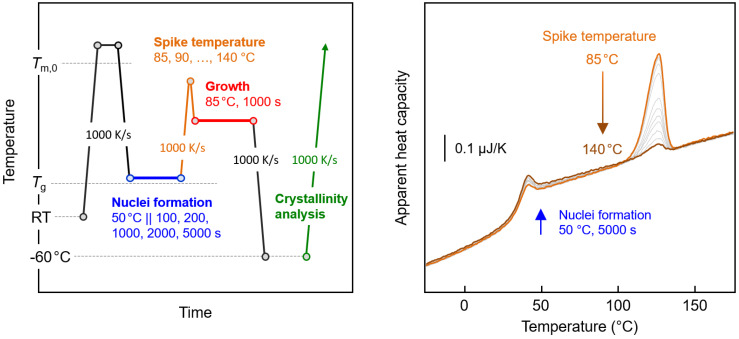
Temperature–time profile for the analysis of the thermal stability of crystal nuclei formed at 50 °C for different times (**left**). FSC heating curves after subjecting the polymer to nucleation at 50 °C for 5000 s, followed by heating to a pre-defined maximum temperature between 85 (orange curve) and 140 °C (brown curve), and the growth of nuclei to crystals at 85 °C for 1000 s (**right**). The gray curves are associated to maximum spike temperatures of 90, 95, 100, 105, 110, 115, 120, 125, 130, and 135 °C.

Figure 3 shows with the left plot the temperature–time profile for the analysis of the thermal stability of crystal nuclei isothermally formed at 50 °C during annealing for different times between 100 and 5000 s (blue segment). Prior research revealed that the nucleation rate is highest at around 50 °C, [27] and variation in the nucleation time may provide information on whether it affects the cluster size distribution. The stability of the nuclei was examined by heating the system at 1000 K/s to different maximum spike temperatures between 85 and 140 °C (orange segments) before probing the nuclei number as a function of the spike temperature by an analysis of the crystal fraction forming within 1000 s at 85 °C, that is, at fixed growth conditions (red segment). We assume that the crystal nuclei formed at 50 °C will “melt” on heating at 1000 K/s to above a nucleus size-dependent critical temperature without prior stabilization/growth. The expected reduction in the nuclei number with the increasing spike temperature is then detected by decreasing the amount of crystals at fixed growth conditions. The assumption of the absence of nuclei-reorganization on heating the system at 1000 K/s is further discussed below, in Section 3.3. As an example, the right graph of Figure 3 shows FSC heating curves after subjecting the polymer to nucleation at 50 °C for 5000 s, heating it to a pre-defined maximum temperature between 85 and 140 °C, and growth of nuclei to crystals at 85 °C for 1000 s. As such, the heating scans represent the green-colored segment in the left plot of Figure 3. The FSC scans reveal a heat capacity increment slightly below 50 °C that is related to the glass transition, superimposed with a small enthalpy recovery peak. Further heating leads to the melting of crystals entirely grown at the growth stage of the experiment. Most important is the detection of decreasing enthalpy of crystallization/melting with increasing maximum spike temperature. The recorded melting peak is largest if the nuclei are directly transferred to the growth temperature of 85 °C, while it becomes smaller on in-between heating to a higher temperature of up to 140 °C, with the decrease of the melting peak area/crystallization enthalpy caused by a lowered number of nuclei.

**Figure 4:**
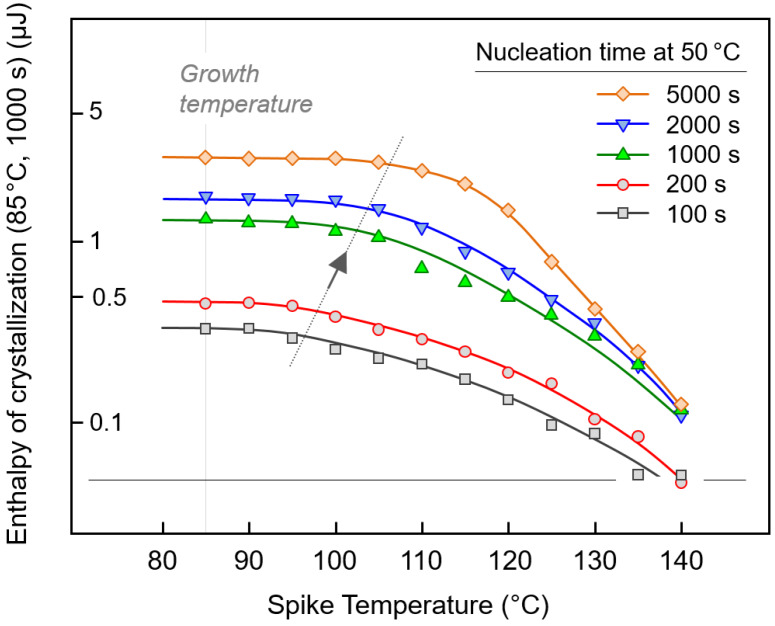
Enthalpy of the crystallization of PBI at 85 °C for 1000 s as a function of the spike temperature on the transfer of nuclei from the nucleation stage to the growth stage (see also left plot in Figure 3). Samples were subjected to nucleation at 50 °C for different times, as indicated in the legend, and the nuclei-transfer heating rate was 1000 K/s. The lines connecting the symbols serve to guide the eye only. The gray line with the arrow emphasizes that the nuclei size distribution changes with the nucleation time such that generation of an increased fraction of larger nuclei shifts the downturn in the curves to higher spike temperatures (see) with increasing nucleation time.

Figure 4 shows quantitative data of the enthalpy of the crystallization of the PBI at 85 °C for 1000 s as a function of the spike temperature, after nucleation at 50 °C. The different data sets represent samples subjected to nucleation for different times, as indicated in the legend. First, the data reveal that the observed enthalpy of crystallization increases with the time of nucleation due to the increasing number of nuclei; see, for example, the increase of the crystallization enthalpy along the gray vertical line at 85 °C when increasing the nucleation time from 100 s (black square) to 5000 s (orange diamond). In a classical Tammann nuclei development experiment, the growth stage—in this work at 85 °C—is directly approached without intermediate heating to higher temperatures, and plotting the enthalpies of crystallization as a function of the nucleation time provides information about the nucleation kinetics, as reported elsewhere [27]. Second, when reading the individual data sets as a function of the spike temperature, it appears that the enthalpies of crystallization remain constant at an upper plateau and then decrease above a critical value. For example, annealing PBI at 50 °C for 5000 s (orange diamonds) produces populations of crystal nuclei which all survive heating to about 105 °C. However, heating to higher temperatures causes “melting” of populations of crystal nuclei, which are of sub-critical size at their respective spike temperatures; heating to 140 °C leads to almost complete destruction of even the largest and therefore most stable nuclei formed at 50 °C. Third, the data of Figure 4 suggest that the nuclei size distribution changes with the nucleation time. Longer nucleation not only allows for increasing the absolute nuclei number, as detected in an upward shift of the curves, but also for the generation of an increased fraction of larger nuclei. This conclusion derives from the observation that the downturn in the curves shifts to higher spike temperatures (see gray line with arrow) with increasing nucleation time.

### 3.3. Effect of Transfer Heating Rate on Crystal Nuclei Reorganization

**Figure 5:**
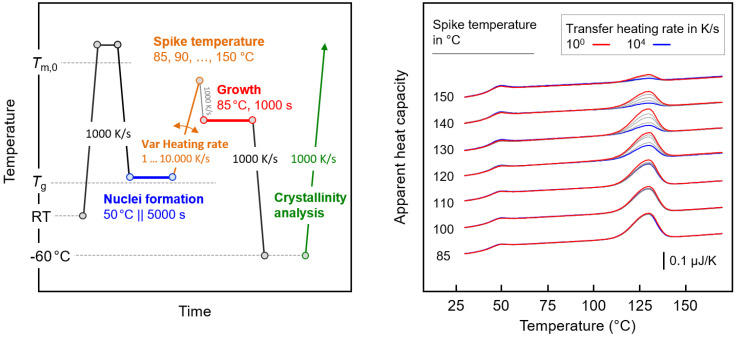
Temperature–time profile for the analysis of the effect of the nuclei-transfer heating rate and spike temperature on nuclei stabilization (**left**). FSC heating curves after subjecting the polymer to nucleation at 50 °C for 5000 s, followed by heating to a pre-defined maximum temperature between 85 and 150 °C at different rates between 1 K/s (red curves) and 10,000 K/s (blue curves), and the growth of nuclei to crystals at 85 °C for 1000 s (**right**).

Figure 5 shows with the left plot the temperature–time profile for the analysis of the effect of the heating rate when transferring the crystal nuclei from the temperature of their formation at 50 °C (blue segment) to different spike temperatures prior to the development temperature of 85 °C (red segment). The crystal nuclei were heated at rates between 1 and 10,000 K/s to different spike temperatures (orange segments) before the analysis of their number via the growth stage (red segment) and subsequent evaluation of the achieved crystallinity by the enthalpy of melting (green segment). The right plot of Figure 5 shows sets of FSC analysis heating scans, associated with different spike temperatures as indicated at the left-hand side of the curves. The red and blue-colored curves refer to transfer heating rates of 1 and 10,000 K/s, respectively. A qualitative inspection of the FSC curves reveals that the transfer heating rate has a negligible effect on the number of crystal nuclei for spike temperatures lower than 100 °C. Exposing nuclei formed at 50 °C to higher temperatures than 100 °C reveals a clear heating rate dependence, such that the nuclei number decreases with increasing heating rate. Furthermore, as expected from the data of Figure 4, even if nuclei reorganization is allowed by slow heating, increasing the spike temperature leads to a lowering of the nuclei number, such that heating to 150 °C causes the destruction of almost all nuclei.

**Figure 6:**
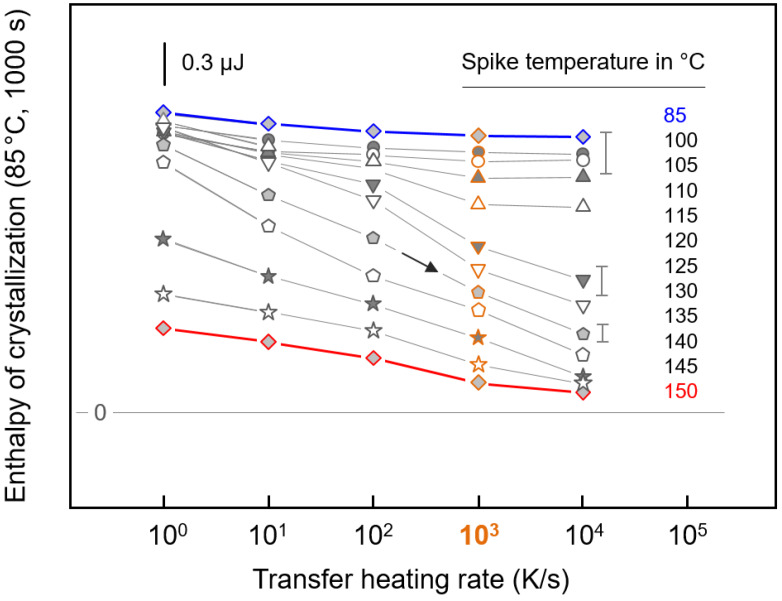
Enthalpy of the crystallization of PBI at 85 °C for 1000 s as a function of the rate of heating crystal nuclei formed at 50 °C for 5000 s to different spike temperatures between 85 °C (blue) and 150 °C (red), as indicated to the right of the various data sets. Data sets for spike temperatures of 85, 100, 110, 120, 130, 140, and 150 °C represent averages of two independent measurements using different samples and FSC sensors, with typical error bars shown to the right. Orange-colored symbols, associated with experiments using a transfer heating rate of 1000 K/s, provide a link to Figure 4, as further explained in the text.

Quantitative data about the transfer heating rate dependence on the nuclei number at the growth-stage temperature, after exposing the system to different maximum temperatures, are shown in Figure 6. Enthalpies of the crystallization of PBI subjected to the thermal profile shown in the left graph of Figure 5, as detected via the enthalpy of melting in the FSC analysis heating scans (see right graph of Figure 5), are plotted as a function of the transfer heating rate, with the different data sets representing samples exposed to different spike temperatures. It is worth repeating that the suppression of the formation of crystal nuclei prior to a crystallization experiment at 85 °C—that is, at the selected growth-stage temperature—does not allow significant crystallization within 1000 s (see right plot in Figure 2). In other words, the detection of the non-zero enthalpy of crystallization at the growth stage in experiments with samples subjected to the thermal profile shown in the left plot of Figure 5 indicates the presence of crystal nuclei formed prior to the growth stage in the nucleation step. Heating crystal nuclei formed at 50 °C for 5000 s at different rates directly to the growth stage (blue symbols/lines) provides the information that a large number of nuclei with a critical size corresponding to the growth-stage temperature of 85 °C were formed. Possible growth/stabilization during heating from 50 to 85 °C, therefore, does not influence the achieved crystallinity at the growth stage.

In-between heating to spike temperatures lower than about 110 °C has only a negligible effect on the change in the crystal-nuclei populations evident at 50 and 85 °C, as only a minor decrease in the enthalpy of crystallization is observed there. This result agrees with data shown in Figure 4, with the orange-colored data points collected from samples subjected to the same nucleation history (50 °C, 5000 s). In the experiments in Figure 4, a transfer heating rate of 1000 K/s was employed, highlighted in Figure 6 by similar orange color-coding. Heating to slightly above 100 °C does not cause a significant reduction in the nuclei number; as well, no effect of the transfer heating rate was observed. For spike temperatures higher about 110 °C, the nuclei number depends on the transfer heating rate, such that it continuously decreases with increasing heating rate (see black arrow); only for data collected up to spike temperatures of 115 °C (open upward triangles) is a lower plateau reached on heating faster, at about 1000 K/s. The decrease in the nuclei number with increasing heating rate is caused by the suppression of their “reorganization”. As such, crystallization enthalpies obtained at a high transfer heating rate reveal information about the relative number of nuclei with a size equal to or larger than the critical size at the spike temperature. In contrast, the slow heating of nuclei of initially (at 50 °C) smaller size than the critical size at the spike temperature allows their growth. For example, when evaluating the experiment with a spike temperature of 135 °C (open/black pentagon symbols), the number of nuclei (at 50 °C) with a size larger than the critical size at 135 °C is negligible (see the data point obtained using a transfer heating rate of 10,000 K/s). Slow heating at 1 K/s, in contrast, allows the growth of a large number of nuclei of initially subcritical size, yielding the same melting enthalpy as on direct heating to 85 °C.

## 4. Conclusions

The main goal of the performed study was the analysis of the thermal behavior of homogenous crystal nuclei of PBI. Crystal nuclei formed at rather low temperatures may disappear/melt on heating to above a critical temperature related to their size, or reorganize/grow to larger nuclei. In the latter case, the “reorganized” nuclei then either “melt” on heating to a higher temperature compared to non-reorganized nuclei or develop into crystals if the selected thermal protocol allows growth. Similarly to crystals, the interplay between direct melting of nuclei on one side and reorganization/stabilization of nuclei during heating on the other side is controlled by the heating rate.

To obtain quantitative information about the thermal behavior of PBI nuclei, Tammann’s two-stage nuclei development method was applied. It involved the formation of nuclei at 50 °C, slightly above the glass transition temperature, and probing nuclei numbers as a function of the transfer conditions (maximum temperature, heating rate) by the evaluation of the developed crystal fraction within a pre-defined time at a growth temperature of 85 °C. For different size distributions of crystal nuclei, as controlled by the nucleation time, a distinct reduction of the nuclei number was detected on heating to maximum temperatures higher than about 90 to 110 °C, with the latter value holding for a longer nucleation time. As such, longer nucleation allows for both increasing the absolute nuclei number and the generation of an increased fraction of larger nuclei. Heating at 1000 K/s to 140–150 °C causes “melting” of even the most stable nuclei. While the direct transfer of crystal nuclei from the nucleation temperature (50 °C) to the growth temperature (85 °C) reveals negligible effect of the transfer heating rate, in-between heating to higher temperatures is connected with distinct nuclei-reorganization above 85 °C on heating slower than 1000–10.000 K/s.

Besides providing specific quantitative information about the characteristics of crystal nuclei of PBI, the performed study emphasizes the importance of the proper design of Tammann’s nuclei development experiment for the analysis of nuclei numbers in general.
